# Scalable Labeling for Cytoarchitectonic Characterization of Large Optically Cleared Human Neocortex Samples

**DOI:** 10.1038/s41598-019-47336-9

**Published:** 2019-07-26

**Authors:** Sven Hildebrand, Anna Schueth, Andreas Herrler, Ralf Galuske, Alard Roebroeck

**Affiliations:** 1 Department of Cognitive Neuroscience, Faculty of Psychology & Neuroscience, Maastricht, The Netherlands; 2 Department of Anatomy & Embryology, Faculty of Health, Medicine & Life Science, Maastricht University, Maastricht, The Netherlands; 3 Systems Neurophysiology, Department of Biology, Technische Universität Darmstadt, Darmstadt, Germany

**Keywords:** Light-sheet microscopy, Brain

## Abstract

Optical clearing techniques and light sheet microscopy have transformed fluorescent imaging of rodent brains, and have provided a crucial alternative to traditional confocal or bright field techniques for thin sections. However, clearing and labeling human brain tissue through all cortical layers and significant portions of a cortical area, has so far remained extremely challenging, especially for formalin fixed adult cortical tissue. Here, we present MASH (Multiscale Architectonic Staining of Human cortex): a simple, fast and low-cost cytoarchitectonic labeling approach for optically cleared human cortex samples, which can be applied to large (up to 5 mm thick) formalin fixed adult brain samples. A suite of small-molecule fluorescent nuclear and cytoplasmic dye protocols in combination with new refractive index matching solutions allows deep volume imaging. This greatly reduces time and cost of imaging cytoarchitecture in thick samples and enables classification of cytoarchitectonic layers over the full cortical depth. We demonstrate application of MASH to large archival samples of human visual areas, characterizing cortical architecture in 3D from the scale of cortical areas to that of single cells. In combination with scalable light sheet imaging and data analysis, MASH could open the door to investigation of large human cortical systems at cellular resolution and in the context of their complex 3-dimensional geometry.

## Introduction

Cellular resolution 3D volume microscopy of human cerebral cortex is challenging because of the large size of the human brain and the 3-dimensional geometry of the cortex. The 2–4 mm thick cortical sheet is highly curved and packed with billions of neurons, organized in layers each hundreds of micrometers thick. Traditionally, studies on human cortical cytoarchitecture have been performed on sections with a thickness of less than 100 µm. However, thin sections have no clear geometric relation to the curved cortical sheet, mostly slicing it non-orthogonal to the layer organization^1^. Moreover, due to shape distortions and tearing inherent to the sectioning process, serial sections are extremely difficult to align post-hoc to provide valuable 3D maps of cytoarchitecture. Although a recent surge of optical clearing techniques has transformed microscopic 3D imaging of small transgenic or antibody stained rodent brains^2–7^, translation of these techniques to the much larger adult human brain has remained a challenge. More specifically, labeling for cytoarchitectonic characterization of adult formalin fixed brain samples has so far remained out of reach, particularly when it needs to be scalable in terms of time and cost to the scale of human neocortical areas.

Here, we report MASH (Multiscale Architectonic Staining of Human cortex): a novel scalable nuclear and cytoplasmic labeling approach for optically cleared samples. MASH is suitable for 4–5 mm thick archival (i.e. formalin fixed and long-term stored) adult human cortex samples and enables deep 3D optical imaging. MASH consists of two parts: (1) a set of small-molecule fluorescent dyes and cleared tissue cytoarchitecture labeling protocols (MASH dye protocols) and (2) a set of adjustable refractive index matching solutions (MASH RIMS) which provide a less corrosive alternative to commonly used RIMS in solvent-based clearing protocols. For the MASH dye protocols, we identified four small organic compounds: acridine orange (AO), methylene blue (MB), methyl green (MG) and neutral red (NR), previously used as cytoplasmic and nuclear labels in traditional light microscopy studies^8,9^. We developed adapted protocols for their use as fluorescent labels in large cleared human brain specimen: MASH-AO (green spectrum cell-body label), MASH-NR (red spectrum cell-body label), MASH-MB (far-red spectrum cell-body label) and MASH-MG (far-red spectrum cell nucleus label).

In order to apply the MASH dye protocols in a wide range of human cortex samples, the clearing process must be: (1) potent enough to clear highly myelinated adult human brain tissue up to 4–5 mm thickness within reasonably short time, (2) compatible with MASH dye protocols and, (3) applicable to archival samples available from brain banks and other academic and clinical tissue storing facilities. The DISCO family of solvent-based clearing protocols^4,6,10^ have short clearing times and they have been applied to freshly frozen^11^ and formalin fixed^10^ human brain tissue. A minor challenge in these protocols is posed by the refractive index matching solutions (RIMS): dibenzyl ether (DBE) or mixtures of benzyl alcohol (BA), benzyl benzoate (BB) and diphenyl ether (DPE). Their corrosiveness limits the microscope setups which can be used due to the detrimental effects on many microscope objectives. Therefore, we developed an adapted DISCO approach replacing the RIMS with two new MASH RIMS alternatives. We identified the essential oil *trans*-cinnamaldehyde (CA) as a high refractive index (RI) medium, mixable with 2,2′-Thiodiethanol (TDE) or wintergreen oil (WGO) to create TDE/CA and WGO/CA RIMS. TDE has been used in the past in aqueous, buffered solution for RI-matching of CLARITY processed tissue (RI = 1.46), showing compatibility with immunostainings and transgenic labels^12^. The MASH RIMS have the desirable properties of adjustable RI (from 1.52 to 1.62, therefore compatible to the DISCO RI of 1.56), low melting point and low corrosiveness, which make them a suitable alternative RIMS choice. Applied together, the MASH dye protocols and MASH RIMS allow clearing and labeling of thick adult human brain samples and deep volume imaging of cytoarchitecture.

We first describe the MASH protocol for clearing and labeling thick human formalin fixed brain samples. Subsequently, we show that the four MASH dyes are suitable for both point scanned and light sheet fluorescent 3D microscopy in different color bands, and that the signal is well maintained even when imaging very deep into the cleared tissue. To validate MASH as a cytoarchitecture technique, we compare fluorescent MASH labels against more traditional bright-field Nissl stains on thin sections, and show that known cytoarchitectonic layer classifications can be performed in 3D MASH datatsets. Finally, we demonstrate on large archival samples of human visual areas that MASH has the potential to characterize cortical architecture in 3D from the scale of cortical areas to that of single cells when combined with light sheet fluorescence microscopy.

## Results

**Figure 1:**
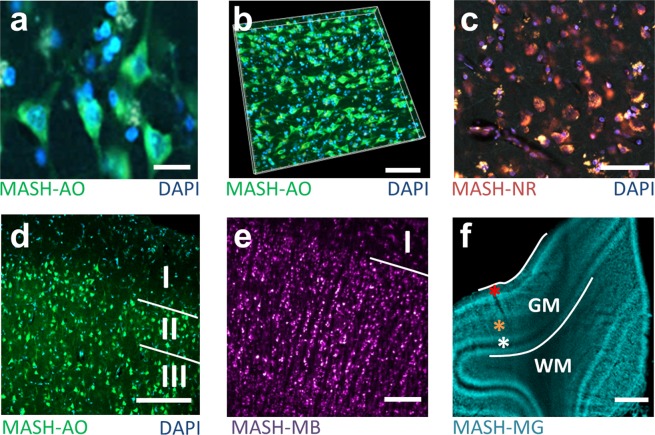
MASH labels human cortical cytoarchitecture in cleared formalin fixed tissue imaged at high resolution and depth. (**a**) Two-photon microscopy (TPM) image of a cleared human neocortex sample stained with MASH-AO for neuronal cell-bodies (green), counterstained with DAPI (blue) for cell-nuclei. (**b**) 3D rendering of the imaging stack in a. (**c**) TPM image of a cleared sample stained with MASH-NR for neuronal somata (red) and DAPI (blue). (**d**) TPM image of MASH-AO and DAPI stain showing the layer I/II and II/III borders (**e**) light sheet fluorescence microscopy (LSFM) of MASH-MB showing the layer I/II border (**f**) low-magnification LSFM imaging of MASH-MG stain showing the entire gray matter (GM), the white matter (WM) transition and cortical layer contrast (red asterisk: layer I; orange asterisk: stripe of Gennari, layer IVb; white asterisk: inner stripe of Baillarger, layer V). Scale bars a: 20 µm; b,c: 50 µm; d: 100 µm; e: 200 µm; f: 1 mm.

**Figure 2:**
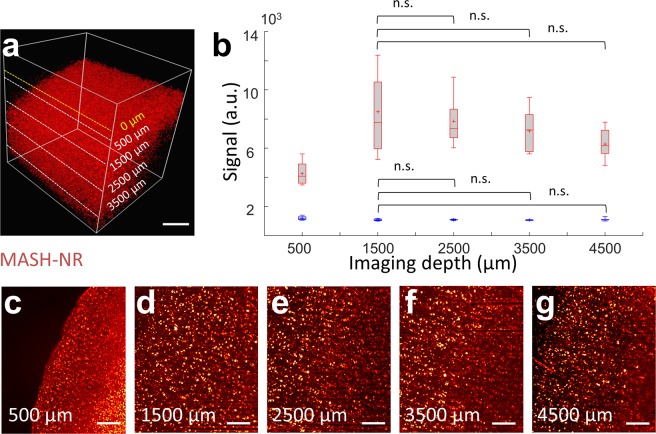
Cell body signal and background tissue signal over depth. (**a**) 3D rendering of 4.5 mm deep (referenced to tissue surface) LSFM imaging stack of MASH-NR stained tissue. Note that for analysis, the start of the tissue was considered as 0 µm depth, not the start of the volume as indicated by the yellow line. (**b**) Mean, median and spread (n = 15) for neuronal cell body signal (red boxplots) and non-neuronal tissue background signal (blue boxplots) at several depths in g. (**c–g**) Images indicated at 500 µm, 1500 µm, 2500 µm, 3500 µm and 4500 µm imaging depth. Scale bars a: 1 mm**;** c–g: 200 µm.

We demonstrated that MASH can clear and label archival adult cortex samples and that cytoarchitecture can be imaged at a variety of wavelengths and magnifications (Fig. 1). Volume imaging can be performed in the green spectrum (MASH-AO, Fig. 1a,b,d; Supplementary Video 1), red spectrum (MASH-NR, Figs 1c, 2) and far-red spectrum (MASH-MB, MASH-MG, Fig. 1e,f; Supplementary Video 5). Green spectrum MASH-AO and red-spectrum MASH-NR are best suited for imaging at sub-micron resolutions using Two-photon microscopy (TPM), because their wavelength allows for two-photon excitation by Ti:Sapphire lasers and for relatively high diffraction-limited resolution. This allows delineating single neuron cell-body morphology (Fig. 1a), high resolution reconstruction in 3D (Fig. 1b) and delineation of cortical layer borders (Fig. 1c,d). Two-photon excitation of far-red spectrum MASH-MB and MASH-MG lies outside the range of Ti:Sapphire lasers and their longer wavelengths permit lower diffraction limited resolutions. Instead, MASH-MB and MASH-MG, as well as red spectrum MASH-NR, are well suited for very deep and large field of view imaging, because of the lower scattering at red and far-red wavelengths. Therefore, imaging with light sheet fluorescence microscopy (LSFM; Fig. 1e,f) can be performed over larger fields of view up to the entire cortical sheet.

### Depth of imaging

Clearing and staining can be performed on 5 mm thick samples in a matter of 10 days. The tissue clearing steps, similar to iDISCO+, lead to a slight tissue shrinkage of ~10% bringing a 5 mm pre-clearing tissue sample to about 4,5 mm post-clearing. Clearing and staining result in imaging of cell bodies with low background and high signal over the full imaging depth (Fig. 2). With the red spectrum MASH-NR dye, neural cell body signal shows only a modest decrease in going from imaging at the first 500–1500 µm of depth to 4500 µm of imaging depth from the surface (Fig. 2b,c–g) whereas tissue background signal stays roughly the same. Unpaired t-tests for difference of mean signal (p-values 1500 µm–2500 µm: p = 0.453; 1500 µm–3500 µm: p = 0.453; 1500 µm–4500 µm: p = 0.0102) and difference of mean background (p-values 1500 µm–2500 µm: p = 0.932; 1500 µm–3500 µm: p = 0.447; 1500 µm–4500 µm: p = 0.565) did not reach significance at a Bonferroni corrected α = 0.05. Note that the color scale in Fig. 2c–g was chosen to illustrate signal and tissue background levels, rather than suppressing background and emphasizing cell body signal. Running image depth-stacks for green spectrum MASH-AO (Supplementary Video 2) and far-red spectrum MASH-MB cell-body labels, also show (Supplementary Videos 3–5) that signal to background contrast is maintained to 3–4 mm of imaging depth. Some signal decrease and slight blurring is visible deeper into the samples and further away from the light sheet illumination side due to an increase in light scattering, a well-known effect in light sheet imaging. Note that the MASH labels could penetrate the complete sample thickness, as a lack of penetration would show as a decrease in staining intensity in the center of the tissue, rather than at the highest depths.

### Validation of MASH dyes with classical stains

**Figure 3:**
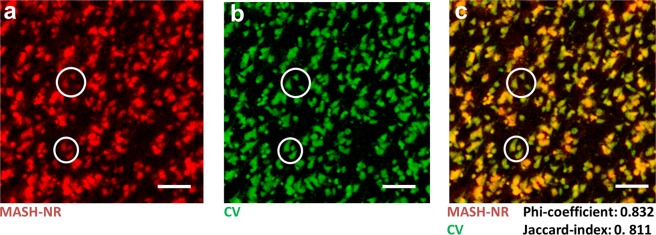
Comparison of MASH-NR and CV on a 50 µm thin section imaged with epifluorescence and bright-field microscopy respectively. (**a**) ROI from the section stained with MASH-NR (pseudocolored in red) and (**b**) with CV (pseudocolored in green). (**c**) Overlay of a and b with corresponding Phi-coefficient and Jaccard-index; circles: corresponding locations. Scale bars: 100 µm.

MASH cell body labelling is well co-localized with standard Nissl stain cresyl violet (CV) in thin sections showing its labeling specificity and suitability for cytoarchitecture characterization (Fig. 3). To quantify pixel-by-pixel overlap of the MASH-NR and CV labels Phi coefficient and Jaccard index were computed for globally optimal thresholds, which gave Phi = 0.832 (at thresholds t_1_ = 53 for CV and t_2_ = 21 for NR, arbitrary units) and Jaccard = 0.811 (at thresholds t_1_ = 41 for CV and t_2_ = 16 for NR, arbitrary units). All non-corresponding pixels at these thresholds were located at the edges of cells labeled by both CV and MASH-NR or in image structures smaller than 4 × 4 pixels (3.2 × 3.2 µm). Performing the same labeling specificity validation against CV for MASH-AO (Supplementary Fig. 1d–f), and MASH-MB (Supplementary Fig. 1h,i), as well as a validation of the MASH-MG nuclear label against DAPI (Supplementary Fig. 1j–l) showed the same high co-localization, with Phi coefficient and Jaccard index in the range 0.6–0.8 (Supplementary Fig. 2). Since the NR, MG and MB molecules can also be used as bright-field stains in thin sections at much (100x–5000x) higher concentrations, we also validated their low concentration fluorescent labeling against the high concentration bright-field labeling in the same thin sections (Supplementary Figure 3), which again show very high co-localization.

### Multiscale LSFM imaging on large samples

**Figure 4:**
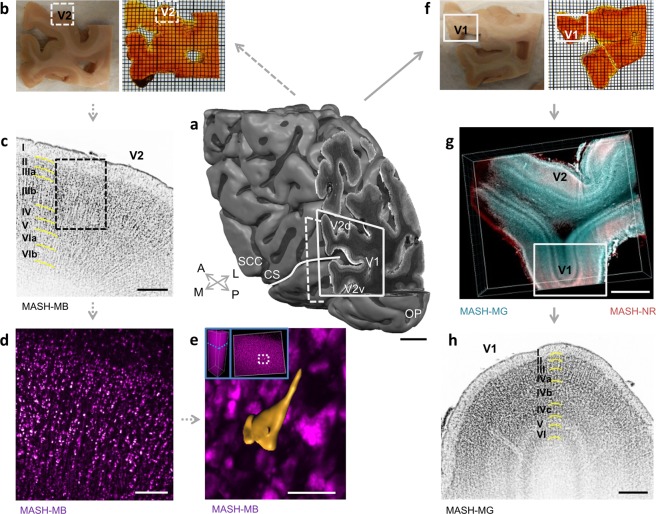
MASH allows characterization of human cortical cytoarchitecture in large formalin fixed samples over a large range of scales. (**a**) 3D MRI reconstruction of the human occipital lobe sample with primary and secondary visual cortex around the calcarine sulcus. V1: primary visual cortex; V2: secondary visual cortex; V2v: ventral V2; V2d: dorsal V2; CS: Calcarine Sulcus (white line); SCC: Splenium of the Corpus Callosum; OP: Occipital Pole; A: anterior; P: posterior; M: medial; L: lateral. (**b**) The anterior 5 mm thick sample (dashed line in a), before (left) and after staining and clearing (right). (**c**) LSFM imaging of the MASH-MB stain in V2 (inverted greyscale map) in the dashed box in b, with cytoarchitectonic layering characterization (left). (**d**) Higher magnification LSFM of the dashed box in c. (**e**) A 3D surface reconstruction of a pyramidal neuron cell body from LSFM data. Inset left: depth position of the cell in the image stack, imaging depth 1066 µm. Inset right: horizontal position in the image stack at the white dashed box. (**f**) The posterior 5 mm thick sample (solid line in a), before (left) and after staining and clearing (right). (**g**) LSFM imaging of dual MASH-NR soma staining and MASH-MG nucleus staining in V1 and V2 in the solid box in f. (**h**) Higher magnification LSFM imaging of the MASH-MG channel in the solid box in g (inverted orientation), with cytoarchitectonic characterization of V1 cortical layering (middle). Thin grid (**b**,**f**): 1 mm. Scale bars: a: 10 mm; g: 2 mm; c,h: 500 µm; d: 200 µm; e: 50 µm.

We demonstrated the efficiency of MASH in high-throughput 3D LSFM characterization of human cortical cytoarchitecture by applying it to large (~40 × 30 × 5 mm) archival samples surrounding the calcarine sulcus (Fig. 4). The samples were large enough to be easily localized in a magnetic resonance imaging (MRI) reconstruction of the entire host occipital lobe and to contain parts of both primary (V1) and secondary (V2) visual cortical areas (Fig. 4a). Clearing was highly effective, rendering the entire 5 mm thick samples transparent with a slight amber-like tint (Fig. 4b,g). Labeling could be achieved over the entire depth of the samples with the far-red MASH-MB cell-body stain for the anterior sample (Fig. 4b–f) and a two-color labeling with the red MASH-NR cell-body stain and the far-red MASH-MG nuclear stain for the posterior sample (Fig. 4g–i).

Multi-spectral LSFM imaging of layered cytoarchitecture could be performed at low magnification over 10–12 mm long stretches of V1 and V2 cortical sheet (Fig. 4h). Higher magnification LSFM produced mesoscale imaging volumes with both the resolution to resolve single neurons and the field of view to contain all layers of the cortical sheet (Fig. 2c,i; Supplementary Video 4). Planes from these volumes allowed for classification of cortical layering and sub-layering, corresponding to a histological reference atlas^13^. They displayed distinctive cytoarchitectonic features such as the cell-poor layers IVb and V in V1 (Fig. 4i) and the large pyramidal cells in layer IIIb of V2 (Fig. 4c). High magnification light sheet imaging (Fig. 4d; Supplementary Videos 2, 3) showed soma morphology features of individual neurons in the context of a deep 3D imaging stack and allowed a coarse surface reconstruction of a pyramidal neuron cell body (Fig. 4e).

## Discussion

The MASH dye protocols allow labeling of thick adult human brain samples and deep volume imaging of cytoarchitecture. The entire MASH protocol for clearing and labeling of 5 mm thick samples takes approximately 10 days. Moreover, MASH dye solution costs are low, less than 1$ per sample. This provides the crucial scalability for the investigation of large human cortical systems and allows application in a wide range of lab environments. MASH is capable of clearing and labeling adult human archival brain samples, even after prolonged storage in formalin (current samples had been fixed for 14 to 30 months), making it applicable to tissue stored in brain banks rather than being limited to fresh or freshly frozen tissue. Additionally, they could be combined with deeply penetrating small-molecule pathology labels to investigate human diseases in their full cytoarchitectonic context as recently demonstrated e.g. by Liebmann *et al*.^11^. Earlier studies using whole-mount cytoplasmic labeling with antibodies or cresyl violet on cleared human brain samples^14,15^ either report over a year of processing time for ~5 × 5 × 5 mm of tissue or report reliable labeling to much lower depth (<  = 1 mm).

MASH was applied here to human occipital (e.g. Fig. 4) and temporal (Supplementary Video 5) neocortical tissue. Human primary visual area is among the most myelinated cortex in the human brain. In addition, even white matter regions showed good transparency after clearing (see Fig. 4b,g; Supplementary Figure g,h). Therefore, it is likely that application of MASH to other brain areas, such as strongly myelinated cerebellum, thalamus or brainstem is likely to yield results of similar quality.

The two low corrosive MASH RIMS can be adapted to various RIs which potentially allows their application in a wider range of optical clearing protocols. Furthermore, samples can be stored long-term in MASH RIMS before imaging because they maintain transparency and fluorescence and do not solidify at low temperatures (2 °C to 7 °C).

Immunohistochemistry has been combined successfully with iDISCO+^11,16^ upon which the clearing approach in MASH is based, showing that it is in principle feasible to combine MASH with antibody labeling. In addition, AO and NR have been used in the past as counterstains on standard histological sections for fluorescent tracers^9^, indicating that they could also act as counterstains in thick, cleared specimen. The usefulness of AO and NR as counterstains for immunohistochemistry or fluorescent proteins might be limited due to the relatively low pH used here. It should be noted however that first, even though the staining gave the best contrast around a pH of 4, it is possible to use especially AO at pH 7.4 as well, which is a more commonly used pH for antibody labeling. Second and more importantly, MG has been demonstrated to work at pH 7.4^8^ and MB has been used at pH 7.4 throughout this work. This renders the application of MG and MB as a counterstaining compatible with immunohistochemistry or transgenic labels likely. The full combination of specific antibodies and MASH labels needs to be investigated in depth in future work.

While antibody labeling would provide highly specific information, their combination with the MASH protocol would likely limit the achievable sample dimensions. Antibodies have limited penetration in thick specimen, mainly caused by the large size of these molecules. Furthermore, their penetration depth seems to be highly variable, depending on the antigen and the antibody. For instance, in a CLARITY based study^15^ penetration over 1–2 weeks varied from 1.2 mm to 5 mm depth, depending on the particular antibody for approximately cube-shaped samples of human brain tissue. In contrast, MASH small molecule dyes can penetrate several millimeters into large samples within days.

A crucial frontier in modern neuroscience is the creation of 3-dimensional cytoarchitectonic reference maps and atlases to provide anatomical context for the interpretation of neuroimaging results at cellular resolution^17–19^. We demonstrated LSFM volume microscopy imaging over a variety of scales with a commercially available light sheet system, both at high resolution and at large field of view. At the resolution limit (~0.5 × 0.5 µm lateral, 4 µm axial) of the employed commercial light sheet microscope the imaging approached cellular resolution, as exemplified by the surface reconstruction of a large pyramidal cell body. However, for reliable single cell reconstructions in the future an improvement in the axial resolution would be needed.

Moreover, light sheet microscope architectures with horizontal side-illumination of the sample can only image the extent of the sample into which the light sheet illumination will penetrate before scattering (several millimeters). The effect of this can be observed e.g. in Supplementary Videos 3–5 in the tissue parts furthest away from the illumination direction of the light sheet. In principle, some effects of scattering can be corrected in post-processing by e.g. flat-field correction or deconvolution. However, in the future, these problems could be fundamentally alleviated by advances of LSFM technology, which would improve both resolution and imaging extent in large samples. Promising in this regard are LSFM architectures such as the dual inverted selective plane illumination microscopy (diSPIM) system geometry^20^ or theta light sheet system^21^, which illuminate and image the sample from above, allowing imaging in thick and arbitrarily wide samples with an automated stage. Moreover, they can achieve high near isotropic resolution, either through multi-view deconvolution or by intersecting two light sheets in a line illumination profile. In order to open new possibilities of cellular level volume imaging of entire human cortical subsystems, such light sheet imaging technology would have to be combined with advances in highly scalable data processing including large-volume image stitching^22^ and automated cell counting^23,24^. If successful, combining the resulting cell-level maps with other imaging modalities, such as MRI (c.f. Fig. 4), could provide correlative multi-modal data on the architecture in the human cortex.

## Materials and Methods

### Human brain tissue

Brain tissue samples were taken from 3 different human body donors (no known neuropathological diseases) of the body donation program of the Department of Anatomy and Embryology, Maastricht University. The tissue donors gave their informed and written consent to the donation of their body for teaching and research purposes as regulated by the Dutch law for the use of human remains for scientific research and education (“Wet op de Lijkbezorging”). Accordingly, a handwritten and signed codicil from the donor posed when still alive and well, is kept at the Department of Anatomy and Embryology Faculty of Health, Medicine and Life Sciences, Maastricht University, Maastricht, The Netherlands.

Brains were first fixed *in situ* by full body perfusion via the femoral artery. Under a pressure of 0.2 bar the body was perfused by 10 l fixation fluid (1.8 vol% formaldehyde, 20% ethanol, 8.4% glycerine in water) within 1.5–2 hours. Thereafter the body was preserved at least 4 weeks for post-fixation submersed in the same fluid. Subsequently, brains were recovered by calvarian dissection and stored in 4% paraformaldehyde in 0.1 M phosphate buffered saline (PBS) for 14–30 months.

For MASH clearing and whole-mount labelling procedures, tissue of an occipital lobe (subject 1, fixation time 14 months), occipital and parietal samples (subject 2, fixation time 30 months) and a temporal lobe sample (subject 3, fixation time 3 months) were used (Supplementary Table 1). All tissue was manually blocked with anatomical trimming blades, then cut into 2 to 5 mm thick slices in coronal orientation and immediately processed.

All methods were carried out in accordance with the relevant guidelines and regulations and all experimental protocols were approved by the Ethics Review Committee Psychology and Neuroscience (ERCPN).

### MASH protocol for clearing and labelling of human brain samples

For clearing of formalin fixed adult brain tissue an adaptation of the iDISCO+ method^16^ was used. The incubation times were adjusted for better clearing of formalin fixed adult human brain tissue and dibenzyl ether (DBE) was replaced by two new MASH refractive index matching solutions (RIMS) adjusted to an RI of 1.56, as described below. The MASH protocol consists of the following steps: (1) sample pretreatment with methanol and bleaching, (2) labelling with MASH dye protocols and (3) Clearing and refractive index matching with MASH RIMS.

### Sample pretreatment with methanol and bleaching

For the pretreatment, samples were dehydrated in ascending concentrations of methanol (VWR International, LLC, 20846.326) in distilled water: 1 h each in 20%, 40%, 60%, 80%, and twice in 100%. Samples were cooled down to 4 °C during the second incubation in 100% methanol. This was followed by bleaching with freshly prepared 5% H_2_O_2_ (1 volume of 30% H_2_O_2_ for 5 volumes of methanol, ice cold) at 4 °C overnight under shaking. After bleaching and re-equilibration to room temperature (RT) the tissue was rehydrated as follows: incubation for 1 h in 80%, 60%, 40% and 20% methanol in distilled water and twice in in 0.1M PBS/0.2% Triton X-100 (VWR International, LLC, 28817.295) respectively. After this, labelling was performed as described below. For all steps, incubation was done in 6 well cell culture plates (Corning Inc., 3516) in a volume of 5 ml/well at RT, unless mentioned otherwise.

### Labeling procedure with MASH dye protocols

Four dyes were identified for cell body (cytoplasm and nucleus) staining or nuclear staining. These dyes, used variously before in animal studies as bright-field or fluorescent stain on histological sections, were investigated as to their suitability as labels of large, cleared adult human specimen for deep fluorescent light microscopy imaging. The four dyes acridine orange (AO), methylene blue (MB), methyl green (MG) and neutral red (NR), see Supplementary Table 4, are referred to as MASH-AO, MASH-MB, MASH-MG and MASH-NR respectively in the context of human cleared tissue labelling protocols, to distinguish them clearly from other prior uses. All MASH labels are small organic compounds with low molecular weight and fluorescent properties which have hitherto not been applied for labelling in thick, cleared human tissue. AO and NR have been variously described before as bright-field or fluorescent Nissl stains on standard histological sections^9^. MG was recently described as an effective, low-cost DNA stain, and applied to chick embryo cryo-sections and whole-mount zebrafish embryos^8^. MB has been used since the early 20^th^ century as a bright-field Nissl stain on thin sections. Here, we optimized the staining protocols for use with thick optically cleared human tissue samples requiring orders of magnitude lower concentrations than bright-field application.

The applied optimized labelling protocol was as follows: All samples were first incubated in freshly filtered solution of 50% potassium disulfite (Sigma-Aldrich, 55777) in distilled water for 1 h at RT. The samples were then washed for 1 h at RT in distilled water. MG stock solution was prepared according to the method of Prieto *et al*.^8^. A 4% aqueous MG (Sigma-Aldrich, 67060) solution was prepared and crystal violet impurities were removed by extractions with chloroform (Sigma-Aldrich, 372978), discarding the lower (violet) phase until no violet tinge could be observed in the lower phase. Stock solution was diluted 1:5000 in 0.1 M PBS with 1% sodium azide (Sigma-Aldrich, S2002) at pH 7.4. Staining procedure for AO and NR was based on the protocols described in Schmued *et al*.^9^. For AO (CarlRoth, 7632.2) and NR (CarlRoth, T122.1) staining, a 1% stock solution in 0.1 M PBS with 1% sodium azide at pH 4 was prepared for each dye. 1% MB (CarlRoth, A514.1) stock solution was prepared in PBS with 1% sodium azide at pH 7.4. Samples for AO, NR and MB staining were incubated in a final concentration of 0.001% in 0.1 M PBS with 1% sodium azide at either pH 4 (AO, NR) or pH 7.4 (MB) for 1 day/mm tissue thickness (2–5 days total) at 4 °C. All staining steps were carried out again in 6 well-plates in a volume of 6 ml/well on a shaker.

### Clearing and refractive index matching with MASH-RIMS

After labelling, samples were washed twice for 1 h in 0.1 M PBS of the respective pH and dehydrated in ascending concentrations of methanol in water: 1 h each in 20%, 40%, 60%, 80%, and twice in 100%. A volume of 5 ml per sample was used for each solution and incubation was performed at RT. Given the corrosiveness of the involved solutions, samples were then transferred into 50 ml incubation tubes made of high-density polyethylene (HDPE). Subsequently, samples were incubated for 3 h (2 mm thick samples) or overnight (5 mm thick samples; see Supplementary Table 1) in a mixture of 33% methanol / 66% dichloromethane (DCM, CarlRoth, 8424.2). Remaining methanol was washed out by incubation in 100% DCM twice for 15 min (2 mm thickness) or twice for 1 h (5 mm). 50 ml tubes were filled completely with each solution.

For refractive index matching, cleared (dehydrated and delipidated) samples were incubated overnight at RT (transparency can already be achieved after several hours of incubation depending on sample thickness and the RIMS used) in 25 ml of one of the newly proposed MASH RIMS: (1) WGO/CA: 72% methyl salicylate also known as wintergreen oil (WGO, Sigma-Aldrich, 84332) and 28% *trans*-Cinnamaldehyde (CA, Sigma-Aldrich, C80687), (2) TDE/CA: 62% *2,2*′-Thiodiethanol (TDE, Sigma-Aldrich, 166782) and 38% CA. The RIMS was changed once right before imaging and tubes were turned upside-down several times until no streaks were visible anymore in the fluid before mounting for microscopic imaging.

### Counterstaining cleared and MASH labelled samples with DAPI

Counterstains were performed with 4′,6-diamidino-2-phenylindole (DAPI) to label cell nuclei in cleared samples labelled with MASH-AO and MASH-NR. For DAPI labelling, 100 mg DAPI (CarlRoth, 6843.3) was dissolved in distilled water to prepare a 0.8 mg/ml stock solution. This stock solution was diluted 1:800 into the freshly prepared working solutions at the respective pH, resulting in a final concentration of 1 µg/ml. For labelling, each brain sample was incubated in a volume of 6 ml for 2 or 5 d at 4 °C on a shaker for 2 or 5 mm thick samples respectively. For incubation 6 well-plates were used. For DAPI co-labelling of sections, an incubation time of 15 min at RT was used.

### MASH-RIMS property comparison

For refractive index matching of dehydrated and delipidated human tissue, the properties of several substances and solutions of various RI’s were evaluated for their use as RIMS (Supplementary Table 2). As a previously unexplored substance for RIMS, *trans*-Cinnamaldehyde (CA, Sigma-Aldrich, C80687) was identified, an essential oil with a very high RI of 1.62 and low melting point which is mixable with TDE and WGO.

The following mixtures were evaluated for their clearing capacity of the dehydrated and delipidated adult human brain samples: 80% glycerol in 0.1 M PBS (pH 7.4, RI = 1.44) and mineral oil (Sigma-Aldrich, M5904) with an RI of 1.47, pure TDE (Sigma-Aldrich, 166782) with an RI of 1.52, pure WGO (RI = 1.54, Sigma-Aldrich, 84332), and pure ethyl cinnamate (ECi, RI = 1.56, Sigma-Aldrich, 112372). Furthermore, mixtures of either 72% WGO and 28% CA or 62% TDE and 38% CA with an RI of 1.56 respectively and a solution of 38% TDE and 62% CA with an RI of approximately 1.58 were tested. As a control, 0.1 M PBS was used. To evaluate corrosiveness, the compatibility with selected plastic materials was checked. Several containers made from polystyrene, polypropylene, high-density polyethylene and tetrafluoroethylene were incubated for up to one week with the solutions described above.

The two MASH RIMS TDE/CA (62% TDE and 38% CA, RI = 1.56) and WGO/CA (72% WGO and 28% CA, RI = 1.56) were identified as having the combined desirable properties of an ideal (and adaptable) RI, low photobleaching, low corrosiveness and convenient storage at low temperature (low melting point). These solutions render archival human brain samples highly transparent with a slight remaining amber color (Fig. 2b,g; Supplementary Fig. 6) typical for many solvent-based clearing approaches. The transparency achieved with the recently described ECi^25^ is similar to the WGO/CA and TDE/CA RIMS (Supplementary Fig. 6). However, ECi has a melting point of 6–8 °C and samples cannot be stored in the fridge once immersed in the liquid. Overall, the TDE/CA RIMS was preferred, because it is even less corrosive for plastic equipment than WGO/CA and had better properties for long-term cold storage (low melting point) than ECi.

### Standard histological sectioning

For the optimization of the final staining protocol (described above) as well as for the validation experiments, standard histological sections of the same brain tissue were used. Therefore, manually cut blocks of human neocortical tissue were sectioned on a vibratome (VT1200 S, Leica Mikrosysteme Vertrieb GmbH, Wetzlar, Germany) into 50 µm thick sections.

### Optimization of staining conditions for MASH dye protocols

Staining conditions for MASH-AO and MASH-NR, in terms of pH and pretreatment were optimized for maximum contrast (Supplementary Figs. 4 and 5). Before staining, sections were incubated in either 50% freshly filtered potassium disulfite solution for 15 min, dehydrated and delipidated in 70%, 100% and 70% methanol for 5 min each or treated first with potassium disulfite and then methanol. Control sections were incubated in 0.1 M PBS for 30 min (Supplementary Figs. 4 and 5). Sections were then washed for 5 min in distilled water and stained with AO or NR in the respective dye solutions described above for 15 min and washed in 0.1 M PBS of the respective pH for 5 min twice. After that sections were mounted in Kaiser’s glycerol gelatine (CarlRoth, 6474.1).

### Validation of specificity of MASH dye protocols

Three validation experiments were performed on standard sections. (1) MASH dye protocols MASH-AO, MASH-NR and MASH-MB were compared in the same sections with standard bright-field (BF) stain Cresyl Violet. (2) MASH dye protocol MASH-MG was compared in the same sections with standard fluorescent stain DAPI. (3) MASH dye protocols MASH-NR, MASH-MB and MASH-MG were compared in the same sections with higher concentrations of the same dye imaged as a bright-field stain.

For experiment 1, standard histological sections were pretreated with potassium disulfite solution and stained with MASH dye protocols MASH-AO, MASH-NR and MASH-MB as described. Sections were then dehydrated in 50%, 70% and 100% methanol for 5 min each, delipidated in 66% DCM/33% methanol for 15 min, and washed twice in 100% DCM for 5 min. This was followed by rehydration in 100%, 70%, 50% methanol and twice in 0.1 M PBS of the respective pH for 5 min. Sections were mounted in Kaiser’s glycerol gelatine. For comparison of the MASH dye protocols with bright-field stains, coverslips of the MASH-labelled sections were removed by immersion in warm water after fluorescent imaging. Subsequently, sections were washed in warm distilled water for 5 min. and stained with cresyl violet acetate (AlfaAesar, J64318) by first dehydration in 70% and 100% ethanol for 5 min and rehydration in 70% ethanol and distilled water for 2 min. This was followed by incubation in freshly filtered 50% aqueous potassium disulfite solution for 15 min and two washes in distilled water for 5 min each. For staining a filtered cresyl violet solution of 1.5% in water, 1% acetic acid and 1% 1 M sodium acetate was used. Samples were stained for 5 min and subsequently washed in acetate buffer for 2 min. Differentiation and dehydration was performed in 70%, 96% and 100% ethanol. Finally samples were immersed in xylol for 5 min twice and mounted in entellan^®^ (Supplementary Fig. 1a–i). For experiment 2 on MASH-MG, the same procedures were followed, but sections were co-labelled with DAPI as described above for DAPI counterstaining (Supplementary Fig. 1j–l).

For experiment 3, sections fluorescently labelled with MASH-MB, MASH-MG and MASH-NR were stained with higher concentrations of the same dyes as a BF control. To this end, coverslips were removed and the sections were washed as described above. Sections were then stained in aqueous stock solutions of MB or MG, or in 0.1% of NR in 0.1 M PBS at pH 4 for 5 min respectively. Hereafter, sections were washed 5 min in 0.1 M PBS with a pH of 7.4 (MB and MG) or pH 4 (NR) and differentiated in 70% and 100% ethanol. After two 3 min incubations in xylol, sections were mounted in entellan^®^ and imaged (Supplementary Fig. 3).

### Magnetic resonance imaging

Data acquisitions on the occipital lobe host sample were performed on a research 9.4 T Siemens MAGNETOM scanner (Siemens Healthcare, Erlangen, Germany) according to the methods described in^26^. A 3D multi-echo Gradient echo (GRE) sequence was used to acquire 200 µm isotropic data (Repetition time /Echo times: 45 ms/7.86, 14, 24 and 34 ms, flip angle (FA) = 28 deg, bandwidth (BW) = 120 Hz/px, matrix dimensions = 400 × 400 × 416). Quantitative T_2_* estimation was performed by fitting a mono exponential decay model and a 3D surface reconstruction was created using Brain Voyager QX v2.8.

### Light-sheet and two-photon microscopic imaging

Before two-photon microscopic imaging (TPM), stained and cleared human brain samples were transferred to a glass petri dish, and a cover slip with water drop was placed on top of each sample. For TPM imaging experiments, a two-photon laser scanning microscope (Leica TCS SP5 MP, Leica Mikrosysteme Vertrieb GmbH, Wetzlar, Germany), equipped with a HCX APO L 20x/1.00 W water immersion objective was used. Working distance of the objective was 2 mm and the excitation source was a 140 fs-pulsed Ti:sapphire laser (Chameleon Ultra II, Coherent Inc., Santa Clara, CA, USA), mode-locked at 800 nm. To avoid photobleaching and tissue damage, laser power was kept at 11% resulting in approx. 25–50 mW, at the sample surface. Images and image stacks were acquired with Leica Application Suite Advanced Fluorescence (Leica Microsystems). TPM Image acquisition settings are detailed in Supplementary Table 3.

Light sheet fluoresscence microscopic (LSFM) imaging was performed with the Ultramicroscope II (La Vision Biotech, Bielefeld, Germany), equipped with a SuperK Extreme Supercontinuum white light laser (EXW-12, NKT Photonics, Birkerød, Denmark). The used objective was a MVPLAPO 2X C/0,5 NA objective with dipping cap (Olympus, Japan), a working distance of 5.7 mm and a RI range of 1,33–1,56. The brain samples were fully immersed during the imaging process in 160 ml of imaging medium. TPM and LSFM acquisition settings for all experiments are detailed in Supplementary Table 3.

### Bright-field and fluorescence microscopy of thin tissue sections

For all imaging experiments on (non-cleared) vibratome sections an Olympus BX51WI DSU confocal microscope (Olympus, Center Valley, PA, USA) coupled to a Hamamatsu EM-CCD C9100 camera (Hamamatsu Photonics K. K., Hamamatsu, Japan) was used. The system was equipped with a motorized stage and a LEP MAC 5000 Controller System (Ludl electronic products, Hawthorne, NY, USA). Images were taken with either, an Olympus PlanApo 2x/0.08 NA, 4x/0.16 NA or UPlanSApo 10x/0.40 NA objective (Olympus, Center Valley, PA, USA). Excitation and emission characteristics for all dyes are given in Supplementary Table 2. For acquisition the Stereo Investigator software (MBF Bioscience, Williston, Vermont, USA) was used.

### Microscopy data processing

For image post-processing such as brightness and contrast adjustments, subtraction of background, and thresholding, as well as running depth-stack volume visualization, the open source software FIJI was used^27^. 3D Volume rendering was performed using Bitplane IMARIS. Quantitative signal analysis was performed in MATLAB using the Open Microscopy Environment (OME) MATLAB toolbox. For the cell body and tissue background signal analysis variation over imaging depth (Fig. 2b), at each depth of 500 µm, 1500 µm, 2500 µm, 3500 µm and 4500 µm from the surface of the tissue, three consecutive data planes were used from the imaging stack for analysis (15 planes total). The middle 50% (in the light propagation direction) of each image plane was used to discard data away from the light sheet waist. At each of the five depths, a total of fifteen regions-of-interest (five per plane, three planes) of 3 × 3 pixels were selected for brightness assessment in both cell bodies and tissue background. The signal for each region-of-interest was taken as the average over the 3 × 3 pixel area. For both cell bodies and tissue background, boxplots were created depicting mean (‘+’ sign), median (box center line), 25th and 75th percentiles (box edges) and 9th and 91th percentiles (whiskers) of the n = 15 region-of-interest signals at each depth. Pair-wise testing for difference in mean cell-body signal at different depths (1500 µm vs. 2500 µm, 1500 µm vs. 3500 µm, and 1500 µm vs. 4500 µm) and mean background signal (at the same depths) was performed by unpaired (two-sample) t-tests with un-equal variance. The 500 µm depth plane was excluded from testing as the sample geometry caused tissue at that depth to be imaged at the furthest distance from the light sheet excitation, experiencing greater signal decay due to scattering. Significance level was set at α = 0.05, Bonferroni corrected over the 6 tests, i.e. α = 0.0083 for each test.

For the pixel-by-pixel overlap of CV label and MASH-NR label in a thin section (Fig. 3), as well as for CV/MASH-AO, CV/MASH-MB and DAPI/MASH-MG overlap (Supplementary Fig. 2) the (inverted) CV and MASH dye images were smoothed (Gaussian filter with sigma = 1 pixel) to remove noise, thresholded, and binarized, and the Phi coefficient (Pearson correlation between binary variables) and Jaccard index (size of the intersecting pixel set divided by the size of the union pixel set) were computed. To obtain pixel-by-pixel overlap scores that are relatively independent of threshold and different signal levels in the data, each of the Phi coefficients and Jaccard indeces were computed for a range of thresholds and the maximum value achieved with a single fixed threshold t_1_ for gold standard image (CV or DAPI) and t_2_ for MASH dye (NR, AO, MB or MG) is reported.

## Supplementary information

ReferencesSupplementary information

Supplementary Video 1

Supplementary Video 2

Supplementary Video 3

Supplementary Video 4

Supplementary Video 5

## Data Availability

The datasets generated and/or analyzed during the current study are available from the corresponding author on reasonable request.
