# Supplementary material for: Scalable Labeling for Cytoarchitectonic Characterization of Large Optically Cleared Human Neocortex Samples
Source: Sci Rep. 2019 Jul 26;9:10880. doi: 10.1038/s41598-019-47336-9 (PMC6659684; doi:10.1038/s41598-019-47336-9)
Supplement: Supplementary file 1 — ReferencesSupplementary information [file 41598_2019_47336_MOESM1_ESM.pdf]

Scalable Labeling for Cytoarchitectonic Characterization of Large Optically Cleared Human Neocortex Samples

Sven Hildebrand[1#], Anna Schueth[1#], Andreas Herrler[2], Ralf Galuske[3], Alard Roebroeck[1*]

1. Department of Cognitive Neuroscience, Faculty of Psychology & Neuroscience, and
2. Department of Anatomy & Embryology, Faculty of Health, Medicine & Life Science,
Maastricht University
the Netherlands

3. Systems Neurophysiology
Department of Biology
Technische Universität Darmstadt
Germany

# contributed equally to this work

* Corresponding author
phone: +31 43-3884039, fax: +31 43-3884125
e-mail: a.roebroeck@maastrichtuniversity.nl

Supplementary Information

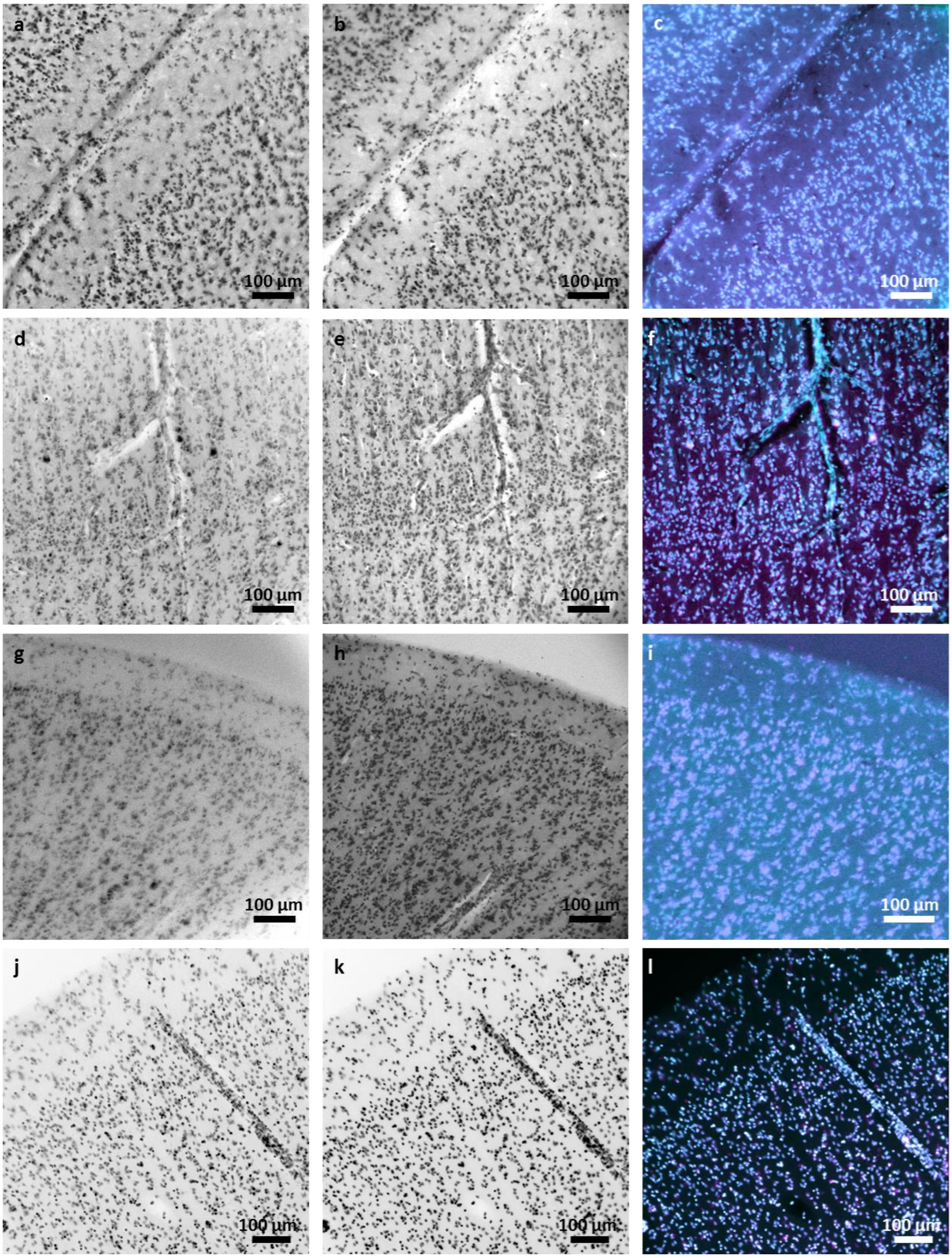

Supplementary Figure 1. Validation of MASH cytoarchitectonic dyes with established labels. Fluorescent images were inverted for better comparison with bright-field images on conventional Nissl staining. (a) MASH-NR stain, (d) MASH-AO stain and (g) MASH-MB stain. (b, e and h) standard bright-field Nissl stain with cresyl violet on the same sections as shown in a, d, and g, respectively. (j) Section stained for nuclei with MASH-MG and counterstained with DAPI (k). (c, f, i and l). Overlay of the MASH labels in the first column (a, d, g and j) in magenta and the control stains in the second column (b, e, h and k) in cyan. Note the high congruency of the labelled structures between the MASH dyes and their respective control staining.

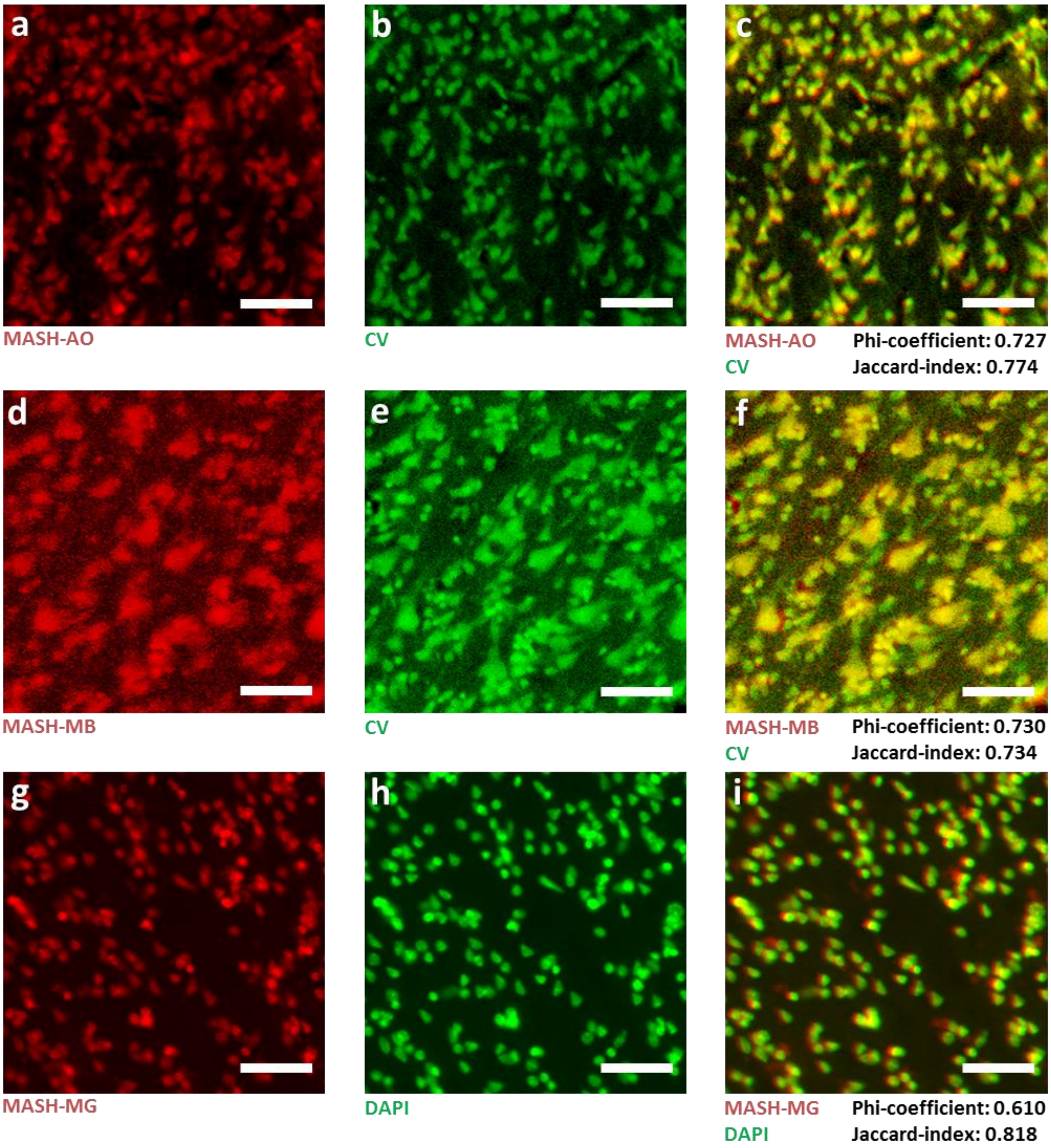

Supplementary Figure 2. Quantitative assessment of co-localization between MASH stainings and conventional staining protocols for Nissl and nuclei. All panels show magnified regions from the images in supplementary figure 1. MASH-AO stain (a) and cresyl violet nissl stain (CV, b) show a high degree of co-localization as seen in the overlay (c) and in the pixel-by-pixel overlap as assessed with the Jaccard-index and the Phi coefficient. The same comparison for MASH-MB and CV is shown in (d – f) and for the nuclear labels MASH-MG and DAPI in (g- i). For every panel MASH stainings have been pseudocolored red and the respective control green. In chase of CV, which was imaged in bright field mode, the pixel values have been inverted. Scale bar: 50 µm in all panels.

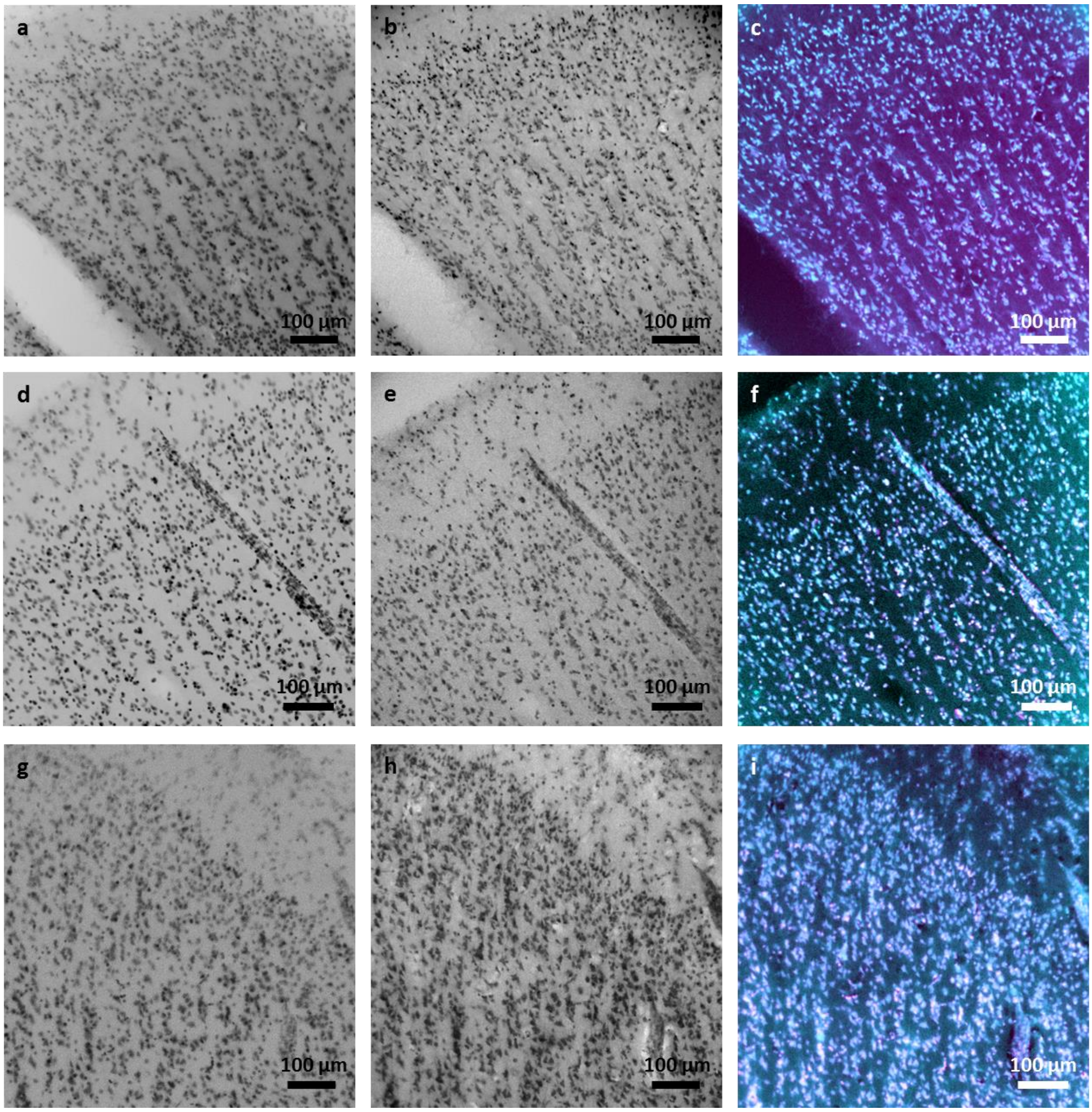

Supplementary Figure 3. Validation of MASH stains with traditional bright-field microscopy using the same dye in much (100x-5000x) higher concentration. Fluorescent MASH label images were inverted for better comparison with bright-field images. (a) MASH-NR stain (b) Bright-field stain with 100x higher concentration of neutral red on the same section. (c) The overlay of MASH-MG in magenta and the bright-field image in cyan shows that the low concentration of the dye used for MASH (magenta) is sufficient to label all structures that are visible in the traditional bright-field stain (cyan). (d) MASH-MG stain (e) Bright-field stain with 5000x higher concentration of methyl green on the same section. (f) The overlay of MASH-MG in magenta and the bright-field image in cyan shows labelling of the same structures. (g) MASH-MB stain (h) Bright-field stain with 1000x higher concentration of methylene blue on the same section. (i) The overlay of MASH-MB in magenta and the bright-field image in cyan again shows a high congruency of labelled structures.

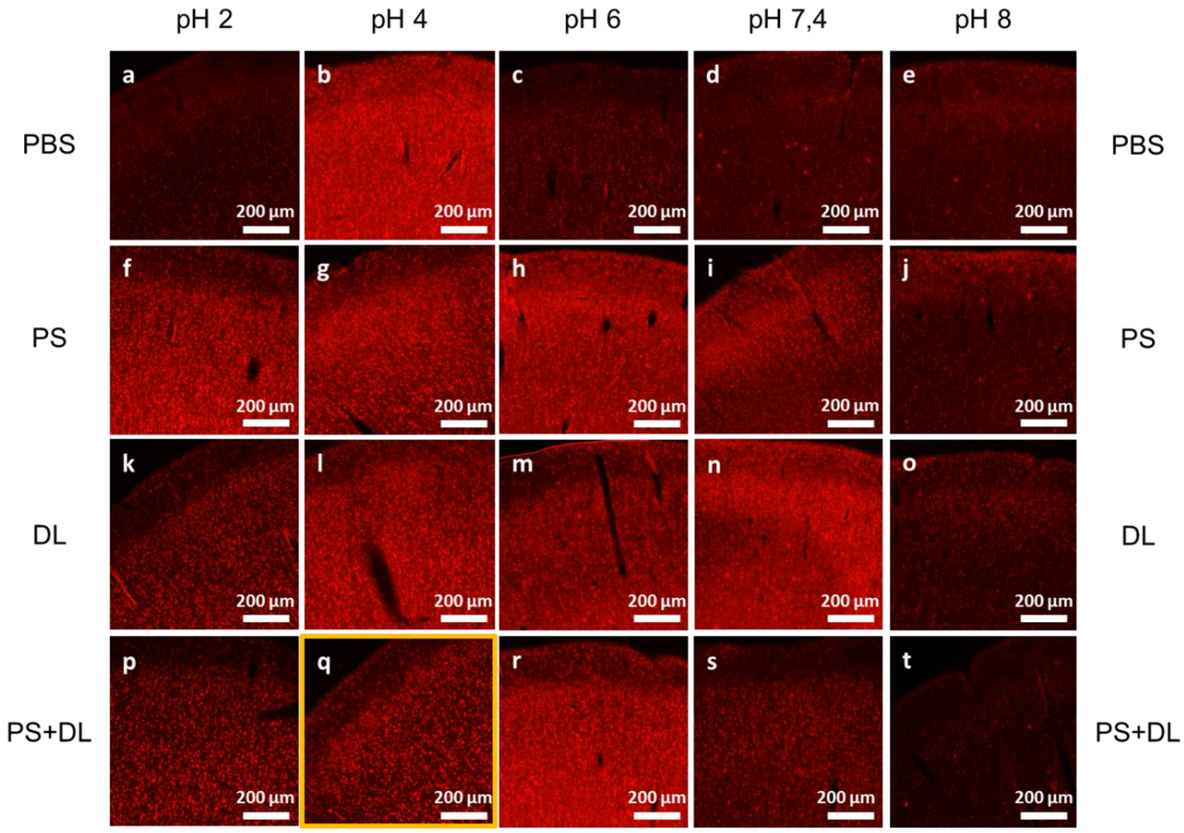

Supplementary Figure 4: Screening for optimal staining conditions for MASH-NR. 50 µm sections of human brain tissue were stained with 0.001 % neutral red and pretreated with several procedures prior to staining. (a – e) Control sections incubated in PBS at respective pH for 30 min. (f – j) Sections were incubated for 15 min in 50 % filtered, aqueous potassium pyrosulfite solution before staining. (k – o) Sections were delipidated with 70 %, 100 % and 70 % methanol in H_2O for 5 min each. (p – t) Samples were first delipidated with 70 %, 100 % and 70 % methanol in H_2O for 5 min each, followed by 15 min incubation in 50 % filtered, aqueous potassium pyrosulfite solution prior to staining. After pretreatment, samples were washed for 5 min in H_2O. All samples were stained in PBS buffered staining solution respectively for 1 h each. pH of the buffered solution was as follows: (a, f, k, and p) pH 2; (b, g, l, and q) pH 4; (c, h, m, and r) pH 6; (d, i, n, and s) pH 7.4; (e, j, o, and t) pH 8. Best results were obtained with the combined delipidation and preatreatment of the tissue with potassium pyrosulfite at pH 4 (q, in orange box).

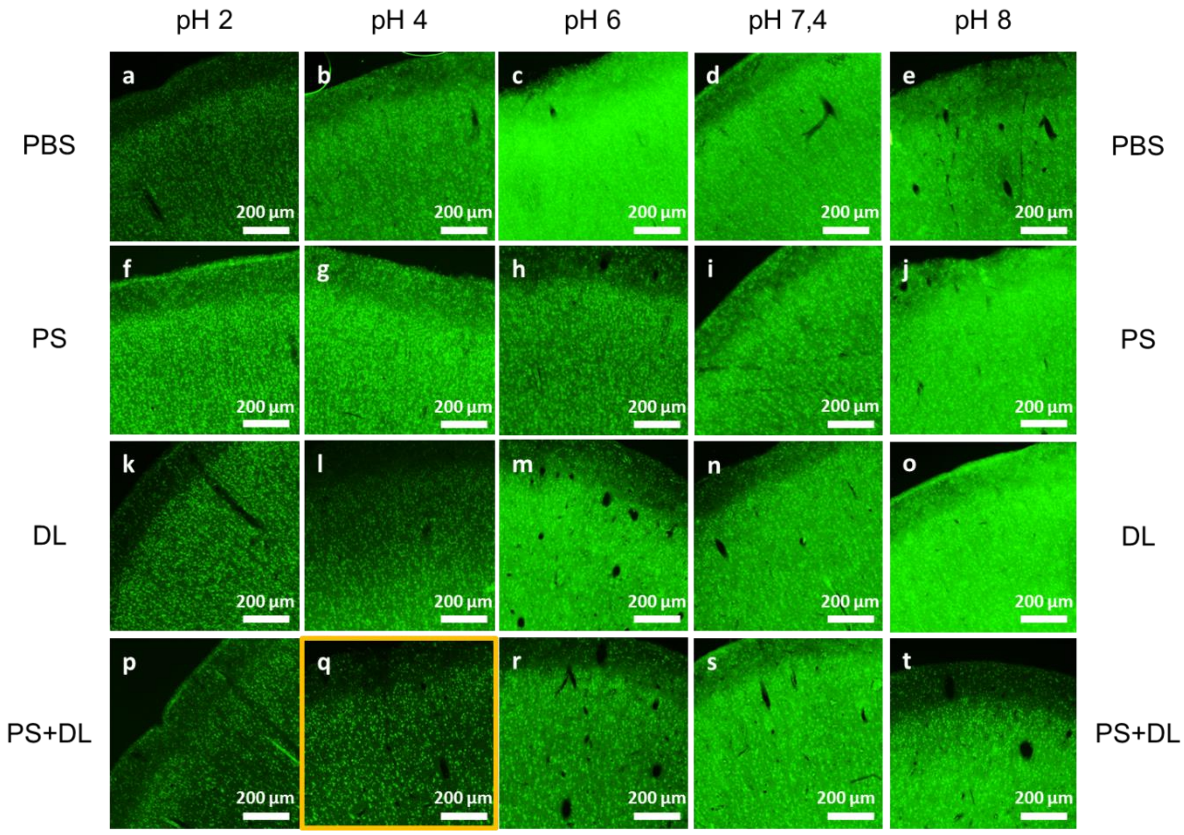

Supplementary Figure 5: Screening for optimal staining conditions for MASH-AO. 50 µm sections of human brain tissue were stained with 0.001 % acridine orange and pretreated with several procedures prior to staining. (a – e) Control sections incubated in PBS at respective pH for 30 min. (f – j) Sections were incubated for 15 min in 50 % filtered, aqueous potassium pyrosulfite solution before staining. (k – o) Sections were delipidated with 70 %, 100 % and 70 % methanol in H_2O for 5 min each. (p – t) Samples were first delipidated with 70 %, 100 % and 70 % methanol in H_2O for 5 min each, followed by 15 min incubation in 50 % filtered, aqueous potassium pyrosulfite solution prior to staining. After pretreatment, samples were washed for 5 min in H_2O. All samples were stained in PBS buffered staining solution respectively for 1 h each. pH of the buffered solution was as follows: (a, f, k, and p) pH 2; (b, g, l, and q) pH 4; (c, h, m, and r) pH 6; (d, i, n, and s) pH 7.4; (e, j, o, and t) pH 8. Staining with pH 2 and 4 resulted in strong labelling independent of pretreatment. Best conditions with very little background were obtained with combined delipidation and potassium pyrosulfite pretreatment at pH 4 (q, in orange box).

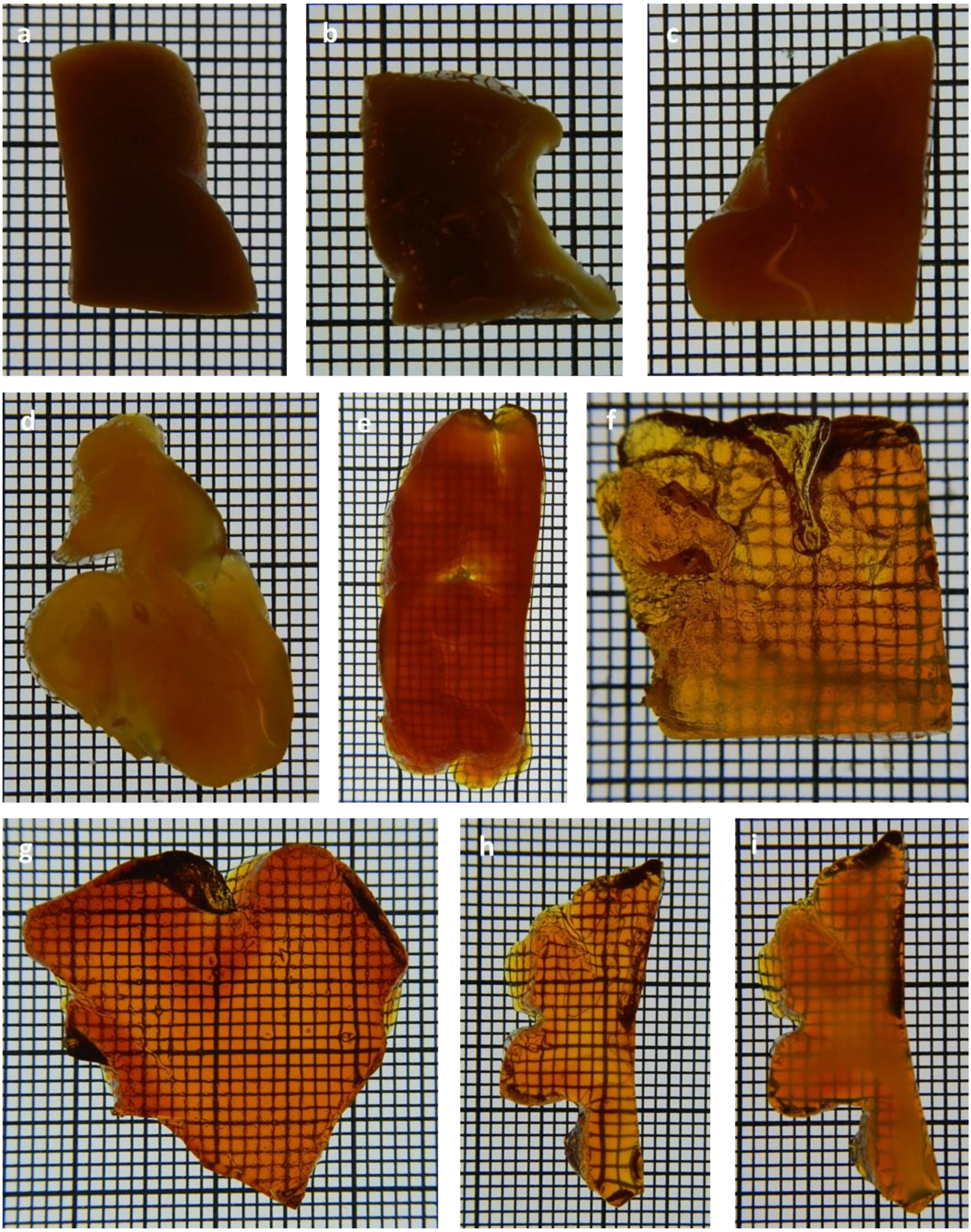

Supplementary Figure 6: Effect of different refractive indices (RI's) on transparency of adult human archival brain tissue cleared with iDISCO+. (a) Sample incubated in PBS after clearing (RI = 1.33). (b) RI = 1.44 with an aqueous solution (80 % glycerol in PBS) (c) RI = 1.47 with a non-aqueous solution (mineral oil). (d) Immersion in 100 % 2,2′-Thiodiethanol (TDE; RI = 1.52). (e) Incubation in pure wintergreen oil (WGO; RI = 1.54). (f – h) Brain tissue incubated in different RI matching media with an RI of 1.56, all of which provide good transparency with a slight amber color. (f) Ethyl cinnamate (ECi). (g) Incubated in a mixture of 72 % WGO and 28 % *trans*-cinnamaldehyde (WGO/CA) (h) 62 % TDE and 38 % CA (TDE/CA). (i) Incubation in 38 % TDE/ 62 % CA solution with a RI of 1.58. Even slight mismatches in RI (e.g. 0.02 lower, in e, or higher, in i) are seen to lead to drastic reduction in transparency. Thin grid: 1mm in all figures.

Supplementary Table 1. Samples, tissue processing and imaging parameters corresponding to figures and videos.

Fig.	Sample Nr.	Subject	Fixation time [months]	sample thickness [mm]	Dye	Incubation time	pH	Medium	Modality	Exc.	Em.	Magnification
1e, 4b–e, Suppl. Video 3, 4	89	Subject 1	14	5	MB	5	7.4	TDE/CA	LSFM	630	680	12.6x, 5x
1f, 4 f-h	91	Subject 1	14	5	NR, MG	5	4	TDE/CA	LSFM	560, 630	620, 680	2x, 4x
2a-g	101	Subject 2	30	5	NR, DAPI	5	4	TDE/CA	LSFM	560	620	12.6x
1a	107	Subject 2	30	2	AO, DAPI	2	4	WGO/CA	TPM	800	550	20x
1b	108	Subject 2	30	2	AO, DAPI	2	4	WGO/CA	TPM	800	550	20x
1d	109	Subject 2	30	2	AO, DAPI	2	4	TDE/CA	TPM	800	550	20x
1c	110	Subject 2	30	2	NR, DAPI	2	4	TDE/CA	TPM	800	600	20x
3a–c, Suppl. 1a-c	111	Subject 2, 1 section	30	0.05	NR, CV	n.a.	4	Slide: Kaiser's glycerol gelatine, Entellan	epifluorescence, bright-field	560	600	10x

Suppl. 1d–f, Suppl. 2a-c	112	Subject 2, 1 section	30	0.05	AO, CV	n.a.	4	Slide: Kaiser's glycerol gelatine, Entellan	epifluorescence, bright-field	488	550	10x
Suppl. 1g-i, Suppl. 2d-f	113	Subject 2, 1 section	30	0.05	MB, CV	n.a.	7.4	Slide: Kaiser's glycerol gelatine, Entellan	epifluorescence, bright-field	630	680	10x
Suppl. 1j-l, Suppl. 2g-i	114	Subject 2, 1 section	30	0.05	MG, DAPI	n.a.	4	Slide: Kaiser's glycerol gelatine, Entellan	epifluorescence, bright-field	630, 405	680, 460	10x
Suppl. 3a-c	115	Subject 2, 1 section	30	0.05	NR	n.a.	4	Slide: Kaiser's glycerol gelatine, Entellan	epifluorescence, bright-field	560	600	10x
Suppl. 3d-f	116	Subject 2, 1 section	30	0.05	MG	n.a.	4	Slide: Kaiser's glycerol gelatine, Entellan	epifluorescence, bright-field	630	680	10x
Suppl. 3g-i	117	Subject 2, 1 section	30	0.05	MB	n.a.	7.4	Slide: Kaiser's glycerol gelatine, Entellan	epifluorescence, bright-field	630	680	10x

Suppl. 4	118	Subject 2, 20 sections	30	0.05	NR	n.a.		8-Feb	Slide: Kaiser’s glycerol gelatine	epifluorescence	560	600	10x
Suppl. 5	119	Subject 2, 20 sections	30	0.05	AO	n.a.		8-Feb	Slide: Kaiser’s glycerol gelatine	epifluorescence	488	550	10x
Suppl. Video 1, 2	120	Subject 2	30	5	AO		5	4	TDE/CA	LSFM	630	680	12.6x
Suppl. Video 5	121	Subject 3	3	5	MB		5	7.4	TDE/CA	LSFM	630	680	2x

Supplementary Table 2. Chemical properties and hazard statements of the different substances tested as RIMS. Corrosiveness refers to the ability of the compound to dissolve plastic materials. Abbreviations: CA = trans-Cinnamaldehyde; TDE = *2,2`*-Thiodiethanol; DBE = Dibenzyl ether; ECi = Ethyl cinnamate; WGO = wintergreen oil/ Methyl salicylate; PBS = phosphate buffered saline. Corrosiveness: 0 = no effect on any plastic tested after 7 days; + = mild to moderate damage on polystyrene after 7 days, no to mild damage on polypropylene after 7 days, no effects on high-density polyethylene and tetrafluoroethylene after 7 days; ++ = moderate to heavy damage on polystyrene after several hours/days, no to mild damage on polypropylene after 7 days, no effects on high-density polyethylene and tetrafluoroethylene after 7 days; +++ = immediate damage on polystyrene, heavy to moderate damage on polypropylene after 7 days, little to no effects on high-density polyethylene and tetrafluoroethylene after 7 days.

Medium	RI	MP [^{o}C]	Corrosiveness
100 % CA	1.622	-9 to -4	++
38 % TDE/62 % CA	1.58	-16 / -9 to -4	++
100 % DBE	1.562	1.5 to 3.5	+++
100 % ECi	1.558	6 to 8	+
62 % TDE/38 % CA	1.56	-16/ -9 to -4	+
72 % WGO/28 % CA	1.56	-8 to -7/ -9 to -4	++
100 % WGO	1.536	-8 to -7	++
100 % TDE	1.5215	-16	0
100 % MO	1.467	n.a.	+
80 % Glycerol in PBS	1.442	n.a.	0
PBS	1.333	n.a.	0

Supplementary Table 3. Image acquisition settings for TPM and LSFM.

Fig.	Sample Number	Dye	Modality	Excitation	Emission	Magnification	Scan field resolution [Pixels]	Scan field size [µm]	Pixel size [µm]	Imaging Depth [µm]
1e	89	MB	LSFM	630/30	680/30	12.6x	2560 x 2160	1318x 1112	0.51	3694
4c	89	MB	LSFM	630/30	680/30	5x	2560 x 2160	3328x2808	1.3	3694
4d	89	MB	LSFM	630/30	680/30	12.6x	2560 x 2160	1318x 1112	0.51	3694
4e	89	MB	LSFM	630/30	680/30	12.6x	2560 x 2160	1318x 1112	0.51	3694
1f	91	NR, MG	LSFM	560/40, 630/30	620/60, 680/30	2x	2560 x 2160	8320x7020	3.25	3800
4g	91	NR, MG	LSFM	560/40, 630/30	620/60, 680/30	4x	2560 x 2160	4160x3510	1.62	3800
4h	91	NR, MG	LSFM	560/40, 630/30	620/60, 680/30	2x	2560 x 2160	8320x7020	3.25	3800
2a - e	101	NR, DAPI	LSFM	560/40	620/60	12.6x	2560 x 2160	1318x 1112	0.51	4700
1a	107	AO, DAPI	TPM	800	550	20x	1024 x 1024	395 x 395	2.6	Single 2D plane from 33 µm stack
1b	108	AO, DAPI	TPM	800	550	20x	1024 x 1024	395 x 395	2.6	33
1d	109	AO, DAPI	TPM	800	550	20x	1024 x 1024	738 x 738	1.4	Single 2D plane
1c	110	NR, DAPI	TPM	800	600	20x	1024 x 1024	434 x 434	2.4	Single 2D plane from 1860 µm stack

Supplementary Table 4. MASH dye molecule properties. Ex.= single photon peak excitation wavelength, Em.= peak emission wavelength

Dye	Labeled Structures	Molecule	PubChem CID	Molecular Formula	Molecular Weight (g/mol)	Ex. (nm)	Em. (nm)
MASH-AO	Cell bodies: Cytoplasm (RNA) & nucleus (DNA)	Acridine orange	62344	$C_{17}H_{19}N_3$	265.36	488	550
MASH-NR	Cell bodies: Cytoplasm (RNA) & nucleus (DNA)	Neutral red	11105	$C_{15}H_{17}ClN_4$	288.779	550	600
MASH-MB	Cell bodies: Cytoplasm (RNA) & nucleus (DNA)	Methylene blue	6099	$C_{16}H_{18}ClN_3S$	319.851	630	700
MASH-MG	Cell Nuclei (DNA)	Methyl green	44134822	$C_{27}H_{35}BrClN_3 \cdot ZnCl_2$	653.24	630	700

Supplementary Video 1

3D rendering of 500 µm deep LSFM image stack of MASH-AO stain in TDE/CA of the superficial cortical layers

Supplementary Video 2

3.4 mm deep LSFM image stack of MASH-AO stain in TDE/CA of the superficial cortical layers. Bottom left: imaging depth in µm. Scale bar (bottom right): 200 µm.

Supplementary Video 3

3.7 mm deep LSFM image stack of MASH-MB stain in TDE/CA of layer I (top right), layer II/III (middle) and layer IV (bottom left) in V2 in inverted grayscale colormap. Bottom left: imaging depth in µm. Scale bar (bottom right): 200 µm.

Supplementary Video 4

3.9 mm deep LSFM image stack of MASH-MB stain in TDE/CA of the entire cortical depth in V2 in inverted grayscale colormap. Bottom left: imaging depth in µm. Scale bar (bottom right): 300 µm.

Supplementary Video 5

3.0 mm deep LSFM image stack of MASH-MB stain in TDE/CA of the entire cortical depth in temporal cortex in inverted grayscale colormap. Bottom left: imaging depth in µm. Scale bar (bottom right): 500 µm.
